# A Cuproptosis–Glycolysis Signature Predicts Prognosis and Highlights AURKA as a Therapeutic Target in ccRCC

**DOI:** 10.1155/humu/6111105

**Published:** 2026-05-09

**Authors:** Chang-yu Ma, Ming-xiao Zhang, Hao-tian Tan, Chong-hao Sun, Shu-zhan Sun, Peng Liu, Jian-feng Wang

**Affiliations:** ^1^ China–Japan Friendship Hospital (Institute of Clinical Medical Sciences), Chinese Academy of Medical Sciences & Peking Union Medical College, Beijing, China, cams.ac.cn; ^2^ Department of Urology, China–Japan Friendship Hospital, Beijing, China, zryhyy.com.cn; ^3^ China–Japan Friendship Clinical College, Peking University Health Science Center, Beijing, China, bjmu.edu.cn

**Keywords:** AURKA, ccRCC, cuproptosis, glycolysis, tumor microenvironment

## Abstract

Cuproptosis, a recently identified form of cell death, is closely linked to glycolysis; however, the mechanistic interplay between these processes in clear cell renal cell carcinoma (ccRCC) remains to be fully elucidated. Utilizing data from the TCGA and CPTAC databases, we developed and validated a cuproptosis‐glycolysis–related gene (CuG) scoring model to investigate its associations with clinical outcomes, tumor immune infiltration, immunotherapy response, and drug sensitivity. Our analysis established a robust 10‐gene risk model with significant prognostic value that effectively stratifies ccRCC patients into distinct high‐ and low‐risk groups exhibiting marked differences in clinical profiles and therapeutic responses. Through integrated bioinformatic analyses alongside in vitro and in vivo experimental validation, we identified AURKA as a key functional regulator within this signature. Beyond promoting tumor cell proliferation, migration, and invasion, AURKA may have a significant role in modulating antitumor immunity. Collectively, by establishing a clinically applicable prognostic scoring system and nominating AURKA as a potential therapeutic target, our study offers translational implications for treatment decision‐making in ccRCC.

## 1. Introduction

Globally, more than 400,000 people are diagnosed with renal cell carcinoma each year, and this number is steadily increasing [1–3]. Clear cell renal cell carcinoma (ccRCC) is the most common subtype of kidney cancer, accounting for approximately 70%–80% of all renal cell carcinoma cases [4, 5]. The development of ccRCC is closely associated with the inactivation of the tumor suppressor gene VHL [6–8]. The loss or mutation of this gene leads to the stable accumulation of hypoxia‐inducible factor [7, 9], which in turn activates a series of downstream genes involved in angiogenesis, cell proliferation, and metabolic reprogramming. Although early‐stage localized ccRCC can achieve favorable outcomes through surgical resection, approximately one‐third of patients present with metastatic disease at initial diagnosis [10, 11]. Therefore, thoroughly elucidating the pathogenesis of ccRCC, exploring novel biomarkers, and identifying effective therapeutic targets are crucial for improving patient prognosis, particularly the quality of life for those with advanced disease.

Metabolic reprogramming is a key feature of ccRCC [12]. Silencing of VHL in most ccRCCs activates hypoxia signaling, enabling tumor cells to adapt to the hypoxic microenvironment by enhancing glycolysis, obstructing carbon flux in the tricarboxylic acid (TCA) cycle, reducing oxidative phosphorylation, and promoting angiogenesis [7, 13–15]. More importantly, even in oxygen‐rich environments, tumor cells tend to favor glycolysis over oxidative phosphorylation, which is a phenomenon known as the Warburg effect [16].

Despite advances in immunotherapy and targeted treatment strategies, the heterogeneity and adaptive resistance of ccRCC remain major clinical obstacles. Therefore, exploring novel cell death mechanisms independent of traditional pathways has become a hot topic in current research. Copper is an essential trace element that plays a vital role in maintaining the normal function of copper‐dependent enzymes and proteins [17]. Tsvetkov et al. first reported that excessive intracellular copper accumulation can induce a unique form of cell death known as cuproptosis by triggering the aggregation of specific lipases and the destabilization of Fe‐S cluster proteins [18]. Notably, the study found that these lipases are all involved in the mitochondrial TCA cycle [19]. This reveals a close connection between copper metabolism and mitochondrial respiration. Previous studies have reported cuproptosis in renal cancer [20–22]. Therefore, we hypothesize that copper may influence patient prognosis by interfering with the expression of glycolysis‐related genes.

We established a risk model comprising 10 genes associated with the cuproptosis‐glycolysis axis. Receiver operating characteristic (ROC) curve and prognostic nomogram analyses demonstrated its strong predictive value for ccRCC prognosis. Furthermore, we explored the model′s relevance to immunotherapy by evaluating its relationship with immune infiltration, immune checkpoints, and drug sensitivity.

## 2. Methods

### 2.1. Data Acquirement

We obtained transcriptomic data from 537 cases of kidney renal clear cell carcinoma (KIRC) and corresponding clinical information from The Cancer Genome Atlas (TCGA) database (https://portal.gdc.cancer.gov), among which 514 samples had complete clinical data. In addition, transcriptomic and clinical data for 103 ccRCC cases were retrieved from the Clinical Proteomic Tumor Analysis Consortium (CPTAC) database (https://portal.gdc.cancer.gov). Cases lacking essential survival or clinical information were removed from the analysis. For single‐cell data analysis, primary ccRCC datasets were acquired from GSE159115 and GSE178481, which comprise 10× single‐cell RNA sequencing data from patients with ccRCC.

### 2.2. Cuproptosis‐Glycolysis‐Related Gene Set Acquisition

786‐O cells were treated with PBS or Cu(II)‐elesclomol (Cu‐ES, 100 nM) for 24 h. This concentration was selected based on our preliminary cytotoxicity assays, which determined the IC50 of Cu‐ES in 786‐O cells to be 126.5 nM. Cells were collected for mRNA extraction and RNA‐seq (NovaSeq X Plus, PE150). DEGs were identified using DESeq2 (FDR *p* < 0.05, |log2*F* *C*| ≥ 1). Glycolysis genes were retrieved from MSigDB (BIOCARTA_GLYCOLYSIS_PATHWAY, HALLMARK_GLYCOLYSIS, KEGG_GLYCOLYSIS_GLUCONEOGENESIS, WP_GLYCOLYSIS_AND_GLUCONEOGENESIS, REACTOME_GLYCOLYSIS), and cuproptosis–glycolysis genes were defined as the intersection of DEGs with this glycolysis gene set.

### 2.3. Construction and Validation of the Cuproptosis‐Glycolysis Signature

Using TCGA‐KIRC as the training cohort and CPTAC for validation, prognosis‐related genes were screened by univariate Cox and Kaplan–Meier (KM) survival analyses. To ensure the comparability of different genes in the model, the mRNA expression data were first normalized via log2(*T* *P* *M* + 1) transformation. A prognostic signature was built using LASSO followed by multivariate Cox regression to generate coefficients, and cuproptosis‐glycolysis score (CuGscore) was calculated as ∑(*β*i × Expi). Patients were stratified into high‐/low‐risk groups by the median training‐set score. Correlation analyses were used to visualize group expression changes. Survival differences and predictive performance were evaluated using survival analysis and time‐dependent ROC, with comparisons to standard clinicopathological factors.

### 2.4. Analysis of the Prognostic Value of the Model

To identify independent prognostic predictors for ccRCC, both univariate and multivariate Cox regression analyses were conducted. Subsequently, a nomogram incorporating the CuGscore along with key clinicopathological variables was developed using the “rms” package (Version 6.2.0) to predict 1‐, 3‐, and 5‐year overall survival. The predictive performance of the nomogram was evaluated using calibration curves and ROC curves.

### 2.5. Biological Function and Pathway Enrichment Analysis

DEGs between the two groups were identified using the criteria of adj. *p* < 0.05 and |log^2^FC| > 1. DEGs were then subjected to Gene Ontology (GO) and Kyoto Encyclopedia of Genes and Genomes (KEGG) enrichment analyses using the “clusterProfiler” package (Version 3.12.0) [23]. Subsequently, Gene Set Enrichment Analysis (GSEA) was performed based on the Hallmark gene sets (Version 4.2.1).

### 2.6. Evaluation of Immunologic Efficacy

To comprehensively evaluate the tumor microenvironment (TME), we employed a multifaceted strategy. First, immune cell infiltration across risk groups was profiled using CIBERSORT [24]. Spearman correlation analysis was used to evaluate associations between CuGscore and immune cell infiltration proportions. Next, expression patterns of immune checkpoint and HLA genes were compared between high‐risk and low‐risk patients. Furthermore, the Tumor Immune Dysfunction and Exclusion (TIDE) algorithm (http://tide.dfci.harvard.edu/) was applied to infer potential differences in sensitivity to immunotherapy [25].

### 2.7. Single‐Cell RNA‐Sequencing Data Analysis

scRNA‐seq data were processed and analyzed using the “Seurat” R package (Version 4.4.0) [26]. Quality control was performed by filtering out cells with nFeature_RNA < 400, hemoglobin > 2%, or mitochondrial content > 30%. Data were normalized using the NormalizeData function, and the Top 2000 highly variable genes were selected for PCA. The Top 40 principal components were used for cell clustering, with batch effects corrected by the Harmony algorithm [27]. Nonlinear dimensionality reduction was performed using UMAP. Cell subtypes were annotated with “SingleR” R package (Version 2.2.0) [28], and malignant cells were identified from epithelia by the CopyKAT algorithm (Version 1.1.0) [29]. The CuGscore for each malignant cell was computed with the AddModuleScore function in Seurat. The top and bottom 5% of malignant cells based on CuGscore were selected for comparative analysis. Cell–cell communication and pseudotemporal trajectories were inferred using “CellChat” (Version 2.1.2) [30] R package and “Monocle” (Version 2.26.0) [31] R package, respectively.

### 2.8. Cell Culture

Human renal cancer cell lines ACHN and 786‐O were obtained from the Cell Bank of the Chinese Academy of Sciences, and the murine Renca cell line was acquired from the American Type Culture Collection. 786‐O, ACHN, and Renca cells were cultured in RPMI‐1640 medium with 10% fetal bovine serum (FBS) and maintained at 37°C in 5% CO_2_.

### 2.9. Knockdown (KD) of AURKA

To generate stable AURKA‐KD models, lentiviral vectors expressing AURKA‐specific short hairpin RNA (shRNA) or nontargeting control shRNA were constructed by Genomeditech Biological Technology. ACHN and 786‐O cells were transduced with the respective lentiviruses and subsequently selected with puromycin (2 *μ*g/mL) for 14 days. For the murine Renca cells, lentiviral vectors expressing mouse Aurka‐specific shRNA were also constructed. The target sequences of all shRNA constructs are provided in Table S1.

### 2.10. Real‐Time Fluorescent Quantitative PCR

Total RNA was isolated using the Animal RNA Isolation Kit (Beyotime, China), reverse‐transcribed to cDNA with the PrimeScript RT Reagent Kit (Takara, Japan), and quantified by RT‐qPCR using SYBR Premix Ex Taq II Kit (Vazyme, China). Relative gene expression was calculated by the 2–*ΔΔ*Ct method and normalized to *β*‐actin. All primer sequences used are listed in Table S2.

### 2.11. Western Blot

Total protein was extracted using a Nuclear and Cytoplasmic Protein Extraction Kit (Beyotime, China) supplemented with phenylmethylsulfonyl fluoride (Servicebio, China) and quantified by BCA Assay Kit (Beyotime, China). After electrophoresis and transfer, membranes were incubated with primary antibodies against AURKA and *α*‐tubulin (Proteintech, China), with *α*‐tubulin as the loading control.

### 2.12. Cell Counting Kit‐8 (CCK‐8) Assay

The viability of 786‐O and ACHN cells from the various treatment groups was evaluated with the CCK‐8 assay. Cells were seeded into 96‐well plates, and absorbance was measured at designated time points after incubation using the CCK‐8 kit (GLPBIO, United States).

### 2.13. Wound Healing Assay

Cell migration was evaluated using a wound‐healing assay. Stable control, sh‐AURKA#1, and sh‐AURKA#2 cells were seeded in 6‐well plates. At full confluence, a scratch was made with a 200 *μ*L pipette tip, cells were washed with PBS and cultured in serum‐free medium. Images were taken at 0 and 24 h, and wound closure was quantified using ImageJ (v1.8.0).

### 2.14. Cell Invasion Assay

Cell invasion was assessed using Transwell chambers (Corning, United States). A serum‐free cell suspension (4 × 10^4^ cells/well) was added to the upper chambers, whereas the lower chambers were filled with RPMI‐1640 medium supplemented with 10% FBS. The assembly was then incubated at 37°C for 24 h. Subsequently, migrated cells were fixed with 4% paraformaldehyde, stained with 0.1% crystal violet, and counted under a microscope.

### 2.15. Cell Cycle Analysis

Cell cycle analysis was conducted in ACHN and 786‐O cells following transfection with sh‐NC or sh‐AURKA#1. After transfection, cells were stained with propidium iodide (PI)/RNase Staining Buffer (KeyGEN BioTECH, China), and their DNA content was quantified by flow cytometry (Beckman FC500, United States). The distribution of cells across different cell cycle phases was analyzed using FlowJo (Version 10.10).

### 2.16. Intracellular Lactate Measurement

Intracellular lactate levels were measured using a Lactate Assay Kit (Solarbio, China) according to the manufacturer′s protocol. Briefly, 786‐O and ACHN cells were harvested 48 h posttransfection, washed with ice‐cold PBS, and homogenized in the extraction buffer. After removing endogenous enzymes and proteins, the supernatants were incubated with the reaction mix for 30 min at room temperature. The absorbance was measured at 570 nm using a microplate reader.

### 2.17. Subcutaneous Tumor Model Construction

For in vivo experiments, 4‐week‐old male BALB/c mice were obtained from Beijing Vital River Laboratory Animal Technology Co. Ltd. and housed under specific pathogen‐free conditions in an Animal Biosafety Level‐III laboratory. Following acclimatization, mice were randomly assigned to two groups (*n* = 5 per group): a normal control (NC) group and a KD group. Each mouse received a subcutaneous injection in the right axillary region with 2 × 10^6^ Renca cells that had been pretransduced with either control or Aurka‐targeting lentivirus.

Tumor growth was monitored every 3 days by measuring the length (L) and width (W) using electronic calipers, and tumor volume was calculated according to the formula: *V* = (*L* × *W* ^2^)/2. After 15 days, the mice were humanely euthanized, and the tumors were harvested, weighed, and processed for subsequent analysis. All animal experiments were reviewed and approved by the Animal Care Review Committee of China–Japan Friendship Hospital (Approval No.: ZRDWLL230135).

### 2.18. Flow cytometry

Subcutaneous tumors were dissociated into single‐cell suspensions using a High‐Activity Tumor Tissue Enzymatic Digestion Kit (RWD, China). Tumor‐infiltrating lymphocytes (TILs) were then isolated via a Percoll density gradient (Solarbio, China). The purified TILs were stained with fluorescently labeled antibodies targeting CD45, CD8, IFN‐*γ*, and TNF‐*α* (BioLegend, United States) for flow cytometry analysis.

### 2.19. Statistical Analysis

Statistical analyses were conducted using GraphPad Prism (Version 10.1.2). Data from three independent biological replicates are presented as mean ± standard deviation (SD). For comparisons between two groups of continuous variables, the Student′s *t*‐test was applied for normally distributed data, and the Wilcoxon rank‐sum test was used for multiple ordered groups. A *p* value

< 0.05 was considered statistically significant, with ∗*p* < 0.05, ∗∗*p* < 0.01, and ∗∗∗*p* < 0.001.

## 3. Results

### 3.1. Acquirement of Cuproptosis‐Glycolysis‐Related Genes

**Figure 1:**
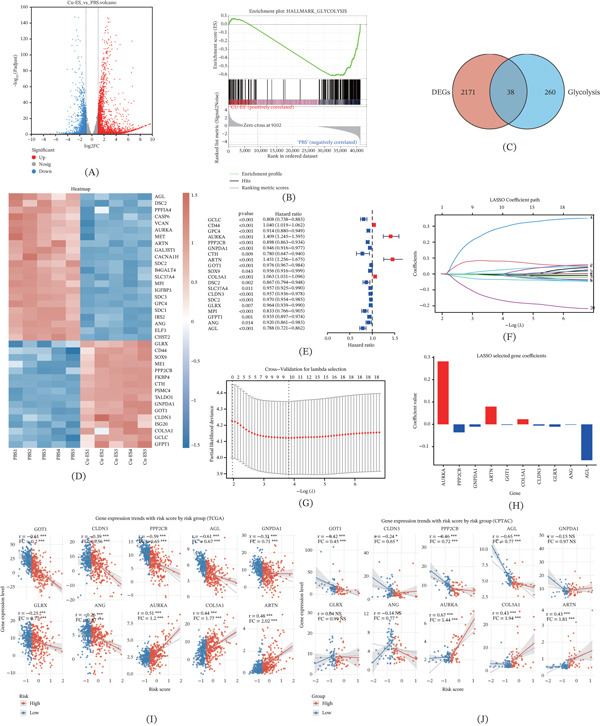
Construction and validation of a prognostic CuG signature for ccRCC. (A) Volcano plot depicting differentially expressed genes (DEGs) between the PBS and Cu‐ES groups. (B) Gene Set Enrichment Analysis (GSEA) of the samples treated with Cu‐ES. (C) The Venn diagram showed the intersection of genes between DEGs and glycolysis‐related genes. (D) The expression patterns of 38 CuG genes are shown in the heatmap. (E) Forest plot for univariate Cox regression analysis of the CuG genes. (F) LASSO coefficient distribution plots displaying the coefficients for each gene. (G) Plots of the cross‐validation error rates. (H) Lasso selected gene coefficients. (I–J) Correlation analysis of 10 signature genes and risk scores. FC, fold changes. ∗*p* < 0.05, ∗∗*p* < 0.01, and ∗∗∗*p* < 0.001.

By screening for DEGs with |log2FC| ≥ 1 and FDR < 0.05, we identified 2209 DEGs between the PBS and Cu‐ES groups (Figure 1A). GSEA revealed significant inhibition of the glycolysis pathway in the Cu‐ES group (Figure 1B). Integration of these DEGs with 298 glycolysis‐related genes from MSigDB yielded 38 cuproptosis‐glycolysis genes (Figure 1C). The corresponding expression heatmap is shown in Figure 1D.

### 3.2. Construction and Validation of Cuproptosis‐Glycolysis Signature

To construct a prognostic signature, we first performed univariate Cox regression on the cuproptosis‐glycolysis‐related genes and identified 20 genes with significant prognostic value (*p* < 0.05; Figure 1E). These genes were subsequently subjected to LASSO‐Cox regression to determine their penalty coefficients (Figure 1F–H), yielding a final 10‐gene signature comprising AURKA, PPP2CB, GNPDA1, ARTN, GOT1, COL5A1, CLDN3, GLRX, ANG, and AGL. The CuGscore for each patient was calculated using the formula: CuGscore = (0.282 × AURKA) + (−0.036 × PPP2CB) + (−0.010 × GNPDA1) + (0.079 × ARTN) + (−0.003 × GOT1) + (0.023 × COL5A1) + (−0.006 × CLDN3) + (−0.01 × GLRX) + (−0.002 × ANG) + (−0.16 × AGL). Patients were stratified into high‐ and low‐risk groups based on the median CuGscore in the TCGA cohort. Further analysis revealed distinct expression patterns among the signature genes: AURKA, ARTN, and COL5A1 were highly expressed in the high‐risk group, whereas the remaining genes showed lower expression (Figure 1I,J).

### 3.3. Clinical Relevance, Independent Prognostic Value, and Nomogram Construction of the CuGscore

To validate the prognostic value of the CuGscore, we utilized the CPTAC cohort as an independent validation set. KM analysis demonstrated that patients in the high‐risk group had significantly shorter overall survival in both the TCGA‐KIRC (*p* < 0.001, Figure 2A) and CPTAC (*p* = 0.01, Figure 2B) cohorts. Furthermore, we assessed the distribution of CuGscores across various clinical subgroups in both the TCGA‐KIRC and CPTAC cohorts (Figure S1). Stratified analysis confirmed the robust prognostic performance of the CuGscore across grade‐ and stage‐stratified subgroups. The ROC analysis further showed that the CuGscore predicted 1‐, 3‐, and 5‐year survival with AUC values of 0.744, 0.702, and 0.720, respectively, in the TCGA‐KIRC cohort (Figure 2C), and 0.810, 0.681, and 0.716 in the CPTAC cohort (Figure 2D).

Figure 2Clinical prognosis analysis of CuGscore. (A–B) The survival curves based on the TCGA‐KIRC and CPTAC cohorts. (C–D) Time‐dependent ROC curves at 1, 3, and 5 years in the TCGA‐KIRC and CPTAC cohorts. (E–F) Forest plot of univariate and multivariate Cox regression analyses assessing the association between risk score and clinical characteristics in the TCGA‐KIRC cohort. (G–H) Forest plot of univariate and multivariate Cox regression analyses assessing the association between risk score and clinical characteristics in the CPTAC cohort. (I) Nomogram constructed using clinical characteristics and CuGscore from the TCGA‐KIRC cohort. (J) Calibration curves for evaluating the nomogram in the TCGA‐KIRC cohort. (K) Time‐dependent ROC curves of the nomogram and clinical features. ∗*p* < 0.05, ∗∗*p* < 0.01, and ∗∗∗*p* < 0.001.

**Figure:**
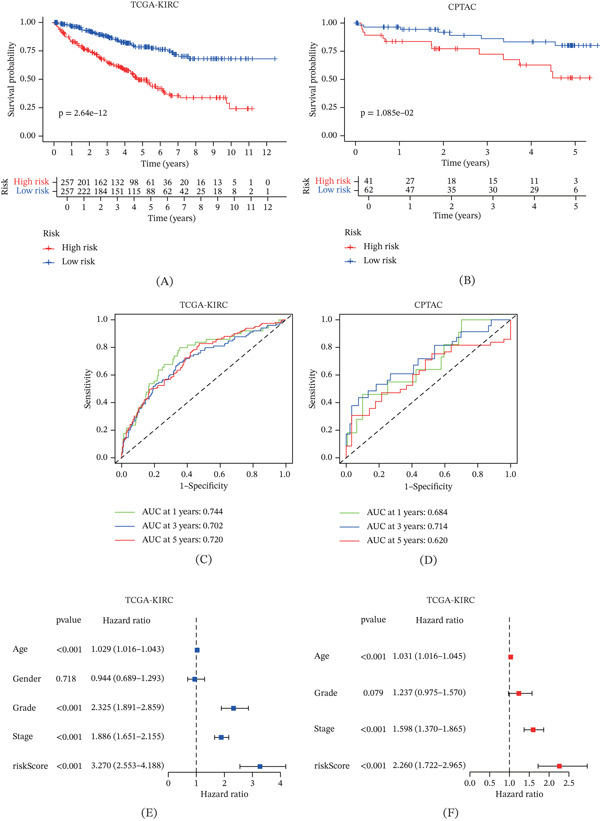
(A)

**Figure:**
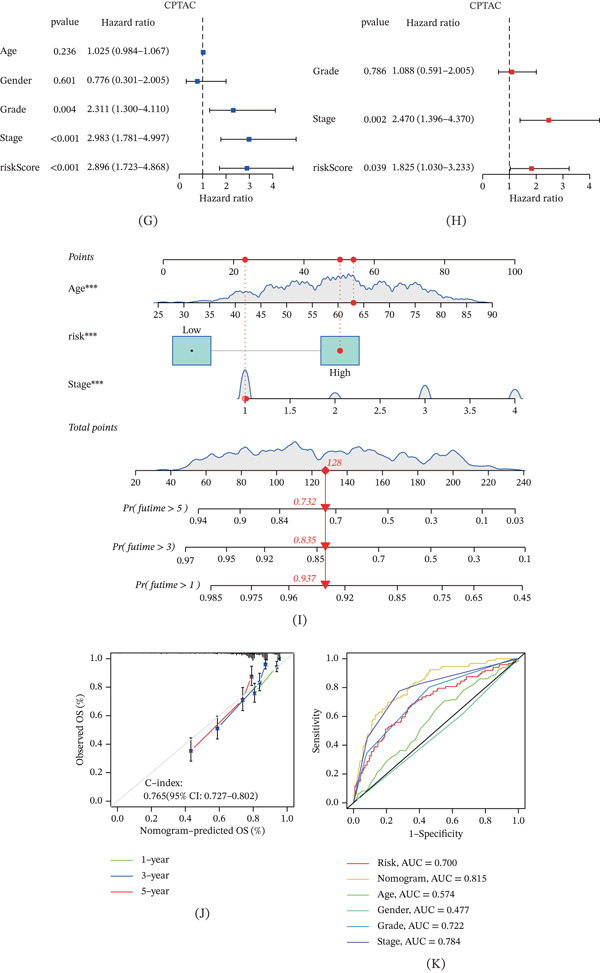
(B)

Univariate and multivariate Cox regression analyses performed on both the TCGA‐KIRC (Figure 2E,F) and CPTAC (Figure 2G,H) datasets identified age, clinical stage, and CuGscore as independent prognostic factors for ccRCC. Integrating these three independent predictors, we developed a prognostic nomogram to estimate 1‐, 3‐, and 5‐year overall survival (Figure 2I). The calibration curves for the nomogram showed excellent agreement between predicted and observed outcomes in the TCGA‐KIRC cohort (Figure 2J). Furthermore, time‐dependent ROC analysis demonstrated that the nomogram achieved a significantly higher AUC compared with any single prognostic factor, underscoring its superior predictive performance (Figure 2K).

### 3.4. Functional Enrichment Analysis

**Figure 3:**
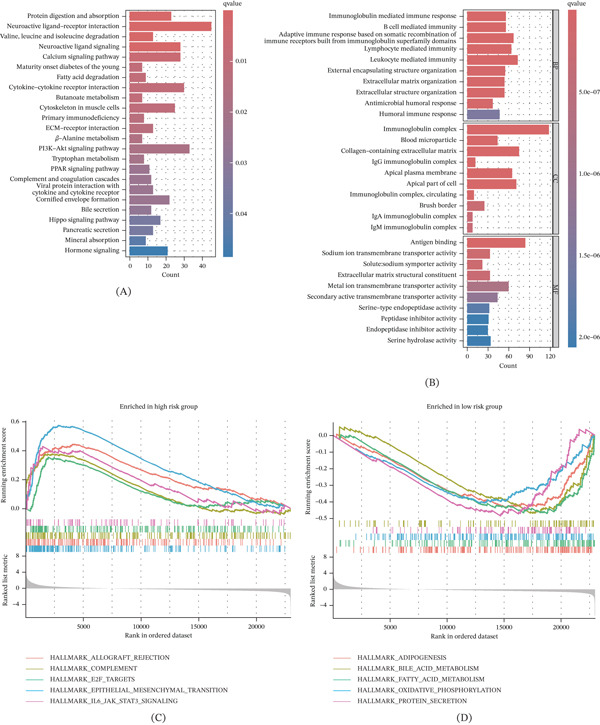
Functional enrichment analysis between risk groups. (A) KEGG functional enrichment analysis of DEGs between risk groups. (B) GO functional enrichment analysis of DEGs between risk groups. (C) GSEA shows enriched pathways in the high‐risk group. (D) GSEA shows enriched pathways in the low‐risk group.

In the TCGA‐KIRC cohort, we identified DEGs between the risk subgroups using the “limma” R package. GO and KEGG analyses revealed that these DEGs were significantly enriched in metabolism‐ and immune‐related biological processes (Figure 3A,B). GSEA further demonstrated distinct pathway activation between the subgroups: the high‐risk group showed significant enrichment of processes associated with poor prognosis, including E2F targets, epithelial‐mesenchymal transition, and IL6‐JAK‐STAT3 signaling (Figure 3C). In contrast, the low‐risk group was characterized by enrichment of oxidative phosphorylation, bile acid metabolism, and fatty acid metabolism pathways (Figure 3D).

### 3.5. Immune Activity Analysis of the CuGscore

**Figure 4:**
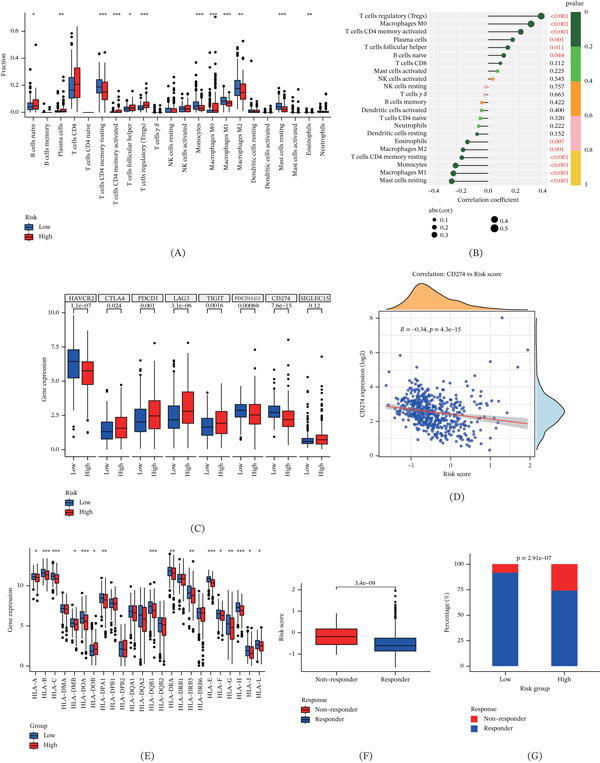
Immune infiltration patterns and immunotherapy response prediction of the CuGscore. (A) Comparison of immune cell infiltration levels between risk groups. (B) Correlation between CuGscore and immune cell infiltration abundances. (C) Differential expression of immune checkpoint genes across risk groups. (D) Correlation analysis of CuGscore with CD274 expression. (E) HLA gene expression profiles stratified by risk group. (F) Comparison of risk scores for different immunotherapy response states. (G) The proportional distribution of immunotherapy responses by different risk groups. ∗*p* < 0.05, ∗∗*p* < 0.01, and ∗∗∗*p* < 0.001.

To explore immunological mechanisms underlying prognostic differences, we compared immune features between risk groups. CIBERSORT estimated infiltration of 23 immune cell types (Figure 4A). Tregs, M0 macrophages, and activated CD4 memory T cells were positively correlated with CuGscores, whereas resting mast cells, M1 macrophages, and monocytes were negatively correlated (Figure 4B). Most immune checkpoint genes were upregulated in the high‐risk group (Figure 4C). Interestingly, we observed that CD274 was negatively correlated with CuGscores (Figure 4D). HLA genes were more highly expressed in the low‐risk group (Figure 4E). TIDE analysis showed higher risk scores in predicted nonresponders (Figure 4F). The results indicated that low‐risk patients were more likely to benefit from immunotherapy, with a higher response rate compared with high‐risk patients (Figure 4G).

### 3.6. Single‐Cell Analysis

To characterize the CuGscore‐associated TME at single‐cell resolution, we analyzed scRNA‐seq data from ccRCC samples. After quality control, we obtained 54,166 high‐quality cells. Quality control and clustering identified eight major cell types, including immune cells (T cells, B cells, macrophages, monocytes, and mast cells), endothelial cells, fibroblasts, and epithelial cells (Figure 5A), with their marker gene expression shown in Figure 5B. GSEA revealed that high‐CuGscore tumor cells exhibited enhanced glycolysis pathway activity (Figure 5C). Pseudotime trajectory analysis further demonstrated that cells with high CuGscores were predominantly enriched at terminal states, whereas low‐scoring cells clustered at root states (Figure 5D). Gene expression heatmaps along pseudotime confirmed upregulation of glycolysis‐related genes (Figure 5E). Cell–cell communication analysis showed that high‐CuGscore malignant cells exhibited stronger interactions and specifically activated SEMA6, CLDN, and CSPG4 signaling pathways (Figure 5F,G), suggesting enhanced proliferative, invasive, and angiogenic capacities. Further analysis revealed that high‐CuGscore malignant cells were enriched with SPP1 and prostaglandin signaling, suggesting a potential mechanism for the immunosuppressive microenvironment.

Figure 5Single‐cell RNA sequencing analysis reveals CuGscore‐associated heterogeneity in ccRCC. (A) UMAP visualization of eight major cell clusters identified in ccRCC samples. (B) Dot plot showing the expression patterns of marker genes across identified cell types. (C) GSEA plot indicating significant enrichment of the glycolysis pathway in high‐CuGscore malignant cells. (D) Pseudotime trajectory analysis showing CuGscore dynamics along malignant cell progression. (E) Heatmap of pathway activities and gene expression patterns along pseudotime in malignant cells. (F) Intercellular communication networks among all identified cell populations. (G) Differential pathway activation patterns across cell populations.

**Figure:**
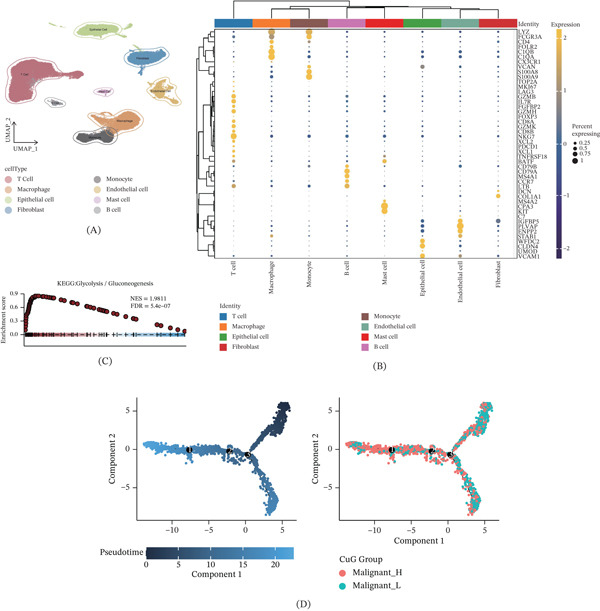
(A)

**Figure:**
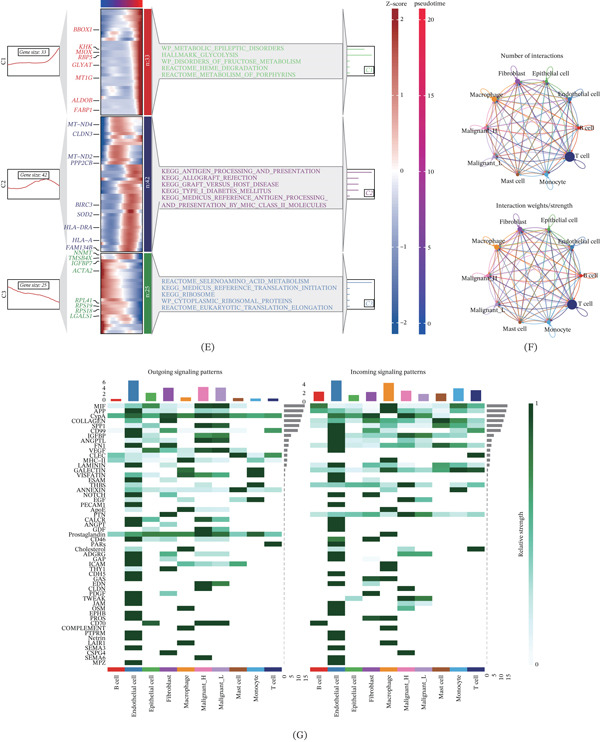
(B)

### 3.7. Identification of AURKA In Vitro and In Vivo

Because AURKA has the highest coefficient, we conducted further research on it. Following Cu‐ES intervention, both 786‐O and ACHN cells displayed a significant downregulation of AURKA at the mRNA and protein levels (Figure 6A,B). The efficacy of AURKA KD was confirmed by Western blotting using two independent shRNAs (Figure 6C). Functionally, AURKA silencing markedly suppressed cell proliferation (Figure 6D), impeded migration in wound healing assays (Figure 6E), and reduced invasive capacity in Transwell assays (Figure 6F). Furthermore, cell cycle analysis revealed that AURKA KD induced S‐phase arrest (Figure 6G). In 786‐O cells, S‐phase cells increased from 27.8*%* ± 0.6*%* in the control group to 33.7*%* ± 1.8*%* in the AURKA KD group. Similarly, in ACHN cells, the S‐phase population increased from 25.6*%* ± 0.8*%* in the control group to 34.4*%* ± 0.8*%* in the AURKA KD group.

Figure 6AURKA promotes tumor progression and modulates antitumor immunity in ccRCC. (A) AURKA mRNA expression in 786‐O and ACHN cells after PBS or Cu‐ES treatment. (B) Western blot analysis of AURKA protein expression following PBS or Cu‐ES treatment. (C) Validation of AURKA knockdown efficiency by shRNA in 786‐O and ACHN cells. (D) Cell proliferation assessed by CCK‐8 assay after AURKA knockdown. (E) Cell migration capacity evaluated by wound healing assay. (F) Cell invasion ability measured by Transwell assay. (G) Cell cycle distribution analyzed by flow cytometry with PI staining. (H) Intracellular lactate measurement. (I) AURKA knockdown sensitizes RCC cells to cuproptosis. (J) Validation of Aurka knockdown in Renca cells. (K–M) Aurka knockdown suppresses tumor growth in mouse subcutaneous tumor models. (N) Production of IFN‐*γ* and TNF‐*α* in tumor‐infiltrating CD8^+^ T cells. ∗*p* < 0.05, ∗∗*p* < 0.01, and ∗∗∗*p* < 0.001.

**Figure:**
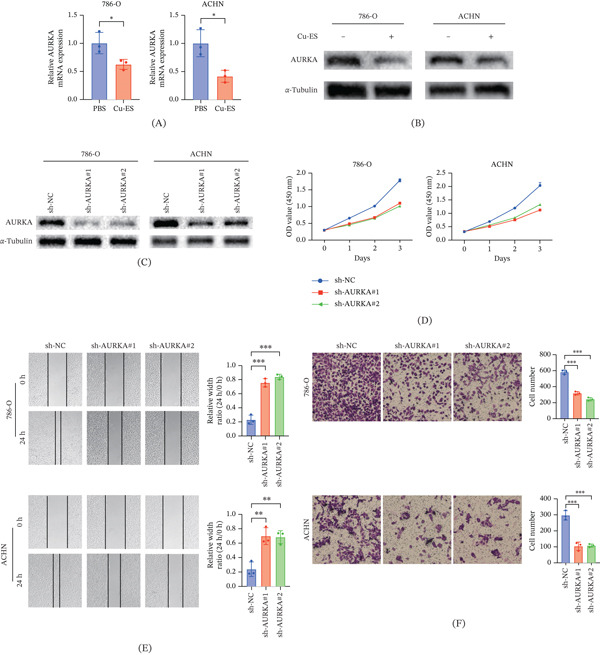
(A)

**Figure:**
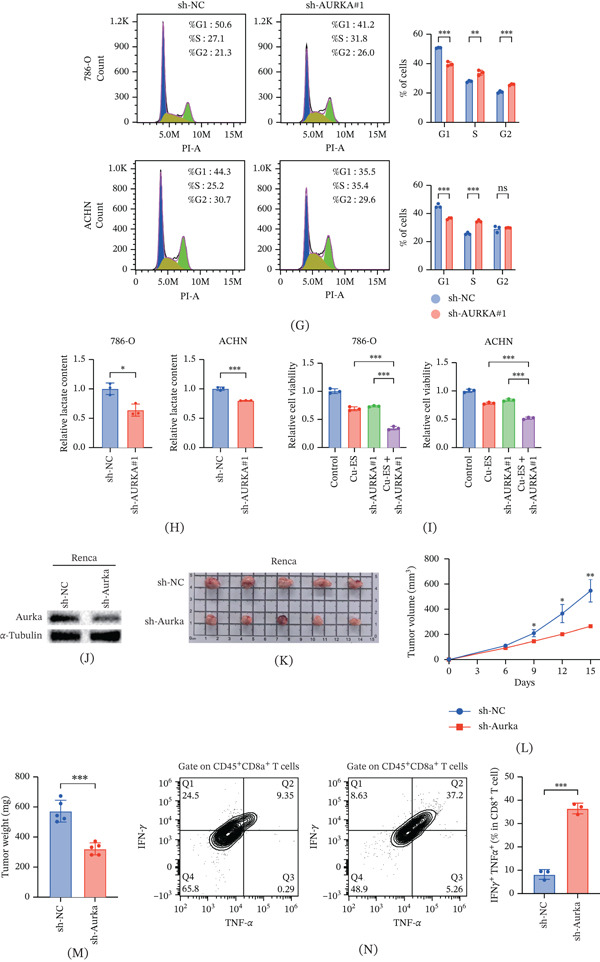
(B)

Our results demonstrated that AURKA KD reduced intracellular lactate levels in renal cancer cells (Figure 6H), indicating a suppression of glycolytic activity. To further explore the biological impact, we assessed the sensitivity of these cells to cuproptosis. Although monotherapy with either Cu‐ES or si‐AURKA resulted in a moderate inhibition of cell viability (20%–30%), their combination elicited a more potent inhibitory effect (Figure 6I). Notably, this observed cytotoxicity surpassed the theoretical additive effect calculated by the Bliss independence model, confirming that AURKA KD effectively sensitizes renal cancer cells to cuproptosis by metabolic reprogramming.

To further validate the oncogenic role of AURKA in vivo, we established subcutaneous tumor models with Aurka‐KD (Figure 6J). The KD significantly suppressed tumor growth and reduced final tumor weight (Figure 6K–M). Concurrently, flow cytometric analysis revealed enhanced antitumor immunity in the TME, characterized by significantly increased production of the cytotoxic cytokines IFN‐*γ* and TNF‐*α* by CD8^+^ T cells (Figure 6N).

## 4. Discussion

Cuproptosis, a recently discovered form of regulated cell death, has been shown to interact with tumor glycolysis [18]. Previous studies have demonstrated that inhibiting tumor glycolysis sensitizes cells to cuproptosis [32–34]. Our results confirm that cuproptosis suppresses glycolysis in ccRCC. As most ccRCC patients exhibit a glycolytic metabolic imbalance due to VHL mutations [7], we hypothesize that the cuproptosis‐glycolysis axis could be a valuable diagnostic and therapeutic tool in ccRCC. Our study highlights the cuproptosis–glycolysis axis as a key determinant of the ccRCC TME and prognosis. We developed and validated a multiomics gene signature that reliably stratifies patients into high‐ and low‐risk groups with distinct survival outcomes. The model underscores the clinical relevance of copper metabolism and may complement current staging to guide personalized surveillance and therapies targeting copper homeostasis.

Although previous studies have developed predictive models based on cuproptosis‐related genes in pan‐cancer [35–37], our approach uniquely derives a 10‐gene signature specifically along the cuproptosis‐glycolysis axis. It robustly stratified patients into high‐ and low‐risk groups with significantly distinct outcomes, as evidenced in both the TCGA‐KIRC training cohort and the CPTAC validation cohort. The signature′s predictive accuracy was consistently strong, with time‐dependent ROC analysis yielding AUC values of 0.744, 0.702, and 0.720 for 1‐, 3‐, and 5‐year overall survival in the TCGA‐KIRC cohort. Our signature demonstrated superior predictive performance, yielding a higher AUC compared with the models developed by Liu et al. [38] (AUC = 0.739, 0.694, and 0.711) and Huili et al. [39] (AUC = 0.709, 0.682, and 0.689). Most importantly, univariate and multivariate Cox regression analyses confirmed that the risk score remained an independent prognostic factor after adjusting for age, stage, and grade. Our CuG signature represents a novel, reliable, and independent prognostic biomarker for ccRCC.

The enrichment analyses demonstrated significant involvement of our signature in immune‐related pathways. This is consistent with the established concept that cuproptosis is a form of immunogenic cell death [40, 41]. GSEA further revealed that the high‐risk group was characterized by marked activation of the cell cycle pathway, indicative of heightened tumor cell proliferative capacity. Concurrent activation of the IL6‐JAK‐STAT3 pathway in this group is implicated in fostering tumor growth and immune evasion [42–44]. In contrast, the low‐risk group exhibited enrichment of metabolic pathways, including oxidative phosphorylation, suggesting a potential suppression of glycolytic activity.

Given that cuproptosis can induce immunogenic cell death and remodel the TME to promote antitumor immunity, we further characterized the immune landscape across risk‐stratified groups. CIBERSORT showed a positive correlation between Tregs and CuGscore, suggesting that higher CuGscore may be associated with increased immunosuppression [45–47]. In contrast, antigen‐presenting cells, including M1 macrophages, mast cells, and monocytes, were more abundant in the low‐risk group, indicating a more active immune environment [48, 49]. The low‐risk group was characterized by reduced expression of most immune checkpoint molecules and elevated expression of HLA genes, which was associated with a more favorable prognosis. Notably, CD274 expression was inversely correlated with CuGscore, further suggesting that low‐risk patients may derive greater benefit from immunotherapy [50, 51]. These findings were corroborated by TIDE analysis and drug sensitivity evaluation, confirming the model′s utility in predicting immunotherapy response and informing treatment selection.

To delineate the molecular mechanisms underlying CuGscore, we performed single‐cell RNA sequencing analysis. GSEA revealed that malignant cells with elevated CuGscores showed significant enrichment in glycolysis‐related pathways. Pseudotime trajectory analysis further demonstrated that these high‐CuGscore cells were predominantly located at terminal states, suggesting a progressive activation of the cuproptosis‐glycolysis axis during tumor evolution. Consistent with this, gene expression dynamics along the pseudotime axis showed upregulation of glycolysis‐associated genes, indicating enhanced malignant potential in advanced tumor cells [52]. Moreover, cell–cell communication analysis revealed that high‐CuGscore malignant cells engaged in more extensive and stronger interaction networks. These cells specifically exhibited activated signaling along the SEMA6, CLDN, and CSPG4 pathways, whose coordinated actions likely drive tumor progression through multiple mechanisms [53–55]. Furthermore, high‐CuGscore malignant cells were enriched with SPP1 and Prostaglandin signaling, which are known to mediate macrophage M2‐polarization and T‐cell inhibition, respectively, suggesting a potential mechanism for the immunosuppressive microenvironment [56, 57].

To experimentally validate our bioinformatics findings, we selected AURKA for further investigation due to its highest prognostic weight in our model. AURKA controls various processes during the cell cycle and is crucial for the assembly and function of bipolar spindles during mitosis and meiosis [58]. In vitro experiments demonstrated that AURKA KD effectively suppressed the proliferation, migration, and invasion of renal cancer cells. Cell cycle analysis further revealed that AURKA depletion induced S‐phase arrest. In addition, consistent with previous findings that AURKA high expression drives tumor glycolysis [59, 60], our study demonstrates that silencing AURKA leads to decreased intracellular lactate production. This shift in metabolic programming subsequently heightens the vulnerability of renal cancer cells to cuproptosis. In vivo validation confirmed that Aurka KD significantly inhibited tumor growth. Notably, Zheng et al. [61] reported that AURKA induces resistance to multiple immunotherapy approaches. Ying et al. [62] similarly demonstrated that inhibiting AURKA fosters an anticancer immune microenvironment and augments the efficacy of antiprogrammed death‐ligand 1 (PD‐L1) therapy. Another investigation found that the Aurora A inhibitor alisertib induced the upregulation of PD‐L1 [63]. We discovered that Aurka knockout enhanced antitumor immunity by promoting CD8 T cell activation and effector function, as evidenced by increased production of IFN‐*γ* and TNF‐*α*. However, the specific mechanisms by which AURKA activates antitumor immunity are still unclear. Collectively, these results establish AURKA as both a promising prognostic biomarker and a functional regulator of antitumor immunity in ccRCC.

## 5. Conclusions

In summary, this study delineates the cuproptosis–glycolysis axis in ccRCC by developing a novel prognostic signature and identifying AURKA as a functional target. The integrated model supports risk stratification and personalized therapy, providing a foundation for future treatments targeting this metabolic network.

## Author Contributions

C.M., M.Z., and J.W. conceived and designed the study. C.M., H.T., and M.Z. drafted the manuscript. C.S. and S.S. collected and organized the data. C.M. and M.Z. carried out all experimental work. H.T., P.L., and J.W. performed data analysis and participated in manuscript revision. C.M., M.Z., and H.T. have contributed to the work equally and should be regarded as co‐first authors.

## Funding

This study was supported by National Natural Science Foundation of China (Grant Number 82303166); Beijing Demonstration Research Ward Construction Project (Grant Number 2022‐YJXBF‐04‐05); National Key Clinical Specialty Construction Project (Grant Number 2020‐QTL‐007); and Elite Medical Professionals Project of China–Japan Friendship Hospital (Grant Number ZRJY2023‐QM30).

## Disclosure

All authors read and approved the final manuscript.

## Ethics Statement

All animal experiments were approved by the Animal Care Review Committee of China‐Japan Friendship Hospital (Approval Number: ZRDWLL230135, Beijing, China) and were conducted in strict accordance with the ARRIVE 2.0 guidelines.

## Conflicts of Interest

The authors declare no conflicts of interest.

## Supporting information


**Supporting Information** Additional supporting information can be found online in the Supporting Information section. Table S1: Target sequences used for gene knockdown. Table S2: Primers used in qPCR. Figure S1: Clinical stratification of the CuGscore. Boxplots comparing the distribution of risk scores across age, gender, stage, and grade between low‐ and high‐risk groups.

## Data Availability

The data that support the findings of this study are available from the corresponding authors upon reasonable request.
