# Supplementary material for: A Cuproptosis–Glycolysis Signature Predicts Prognosis and Highlights AURKA as a Therapeutic Target in ccRCC
Source: Hum Mutat. 2026 May 9;2026:6111105. doi: 10.1155/humu/6111105 (PMC13157311; doi:10.1155/humu/6111105)
Supplement: Supplementary file 1 — Supporting Information Additional supporting information can be found online in the Supporting Information section. Table S1: Target sequences used for gene knockdown. Table S2: Primers used in qPCR. Figure S1: Clinical stratification of the CuGscore. Boxplots comparing the distribution of risk scores across age, gender, stage, and grade between low‐ and high‐risk groups. [file HUMU-2026-6111105-s001.docx]

**Supplementary table 1.** Target sequences used for gene knockdown

| Gene symbol | Target sequence |
| --- | --- |
| AURKA#1 | CCTGTCTTACTGTCATTCGAA |
| AURKA#2 | GAGTCTACCTAATTCTGGAAT |
| Aurka | CCTCATTTCAAGACTGTTAAA |

**Supplementary table 2.** Primers used in qPCR assa

| Gene | Forward primer sequence (5'-3') | Reverse primer sequence (5'-3') |
| --- | --- | --- |
| AURKA | GCTGGAGAGCTTAAAATTGCA | TTTGTAGGTCTCTTGGTATGTG |
| β-actin | CATGTACGTTGCTATCCAGGC | CTCCTTAATGTCACGCACGAT |


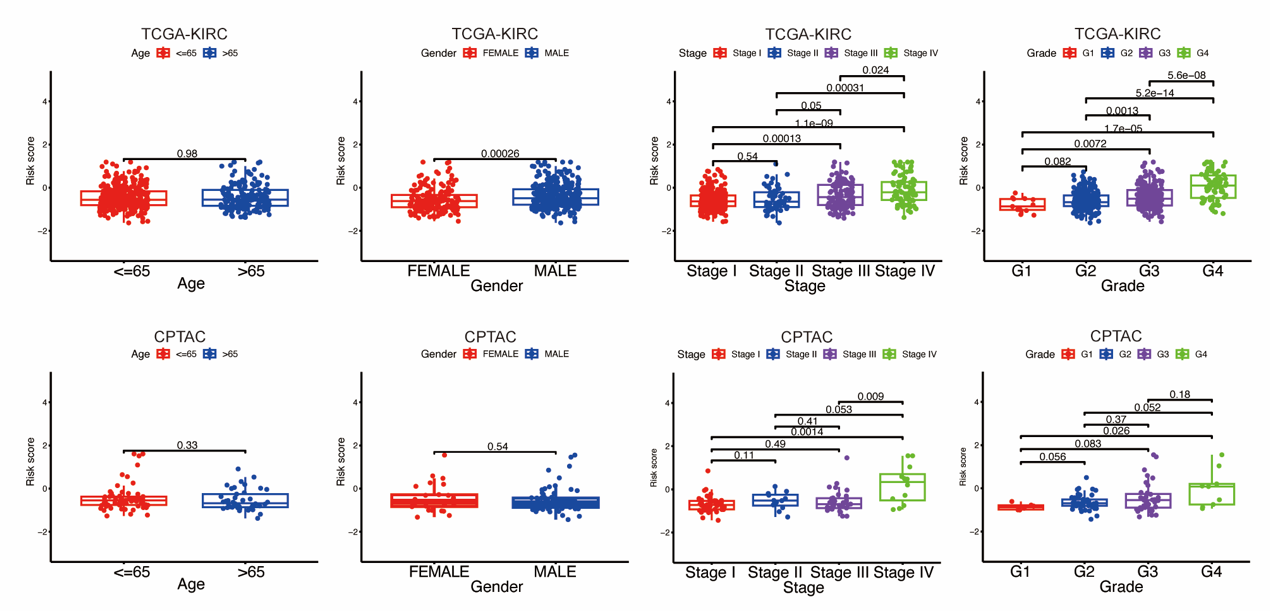


**Figure S1.** Clinical stratification of the CuGscore. Boxplots comparing the distribution of risk scores across age, gender, stage, and grade between low- and high-risk groups.
